# Electroencephalographic Resting‐State Microstates are Unstable in Visual Snow Syndrome

**DOI:** 10.1002/brb3.70374

**Published:** 2025-03-14

**Authors:** Sarah A. Aeschlimann, Antonia Klein, Frédéric Zubler, Christoph J. Schankin, Matthias Ertl

**Affiliations:** ^1^ Department of Neurology, Inselspital Bern University Hospital, University of Bern Bern Switzerland; ^2^ Department of Psychology University of Bern Bern Switzerland; ^3^ Sleep‐Wake‐Epilepsy‐Center, Department of Neurology, Inselspital Bern University Hospital, University of Bern Bern Switzerland; ^4^ Department of Neurology, Spitalzentrum Biel Bern University Biel Switzerland; ^5^ Clinic for Neurology and Neurorehabilitation Luzerner Kantonsspital,University teaching and research hospital Lucerne Switzerland; ^6^ Faculty of Behavioural Sciences and Psychology University of Lucerne Lucerne Switzerland

**Keywords:** EEG microstates, large‐scale network processing, neural network activity, resting‐state EEG, visual snow

## Abstract

**Objective:**

To study large‐scale network processing in visual snow syndrome (VSS) with and without migraine, we applied resting‐state electroencephalographic microstate analysis.

**Background:**

VSS is characterized by a spectrum of visual symptoms, with the main symptom being perceived as flickering dots throughout the visual field. The syndrome is associated with migraine and tinnitus and is considered a network disorder, but the cause and pathophysiology are still largely unknown.

**Methods:**

In this case–control study, resting‐state electroencephalography (EEG) recordings were selected from a cohort of 21 subjects with VSS (8 females, 33 ± 9.56 years, 14 migraines) and 21 matched controls (8 females, 33 ± 11.1 years, 14 migraines). An analysis of parameters between the four canonical microstate Classes A–D was performed.

**Results:**

VSS patients showed an overall shorter duration (*p* = 0.001, Cohen's *d* = −0.48) and lower mean amplitude (*p* < 0.001, Cohen's *d* = −0.76) of microstates compared to the controls. In addition, we found an aberrant syntax of microstate Class A (auditive and visual processing) with more (*p* = 0.001, Cohen's *d* = 0.57) transitions to Class B (visual) and less (*p* = 0.011, *d* = −0.71) to Class C (interoceptive, salience) compared to controls.

**Conclusion:**

Visual snow syndrome (VSS) is a complex disorder, affecting widespread neural network activity. In VSS, electroencephalography (EEG) microstate analysis revealed unstable microstates as well as aberrant transition probabilities, indicating disturbed large‐scale network activity. The analysis of microstate dynamics in VSS complements the results of imaging with high temporal resolution and could contribute to the development of future treatment approaches.

## Graphical abstract

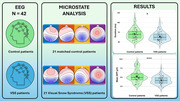

**Abbreviations**
DAN: dorsal attentional network
DMN: default mode network
EEG: electroencephalography
fMRI: functional magnetic resonance imaging
GFP: global field power
*M*
: mean
POI: parameter of interest
rTMS
: repetitive transcranial magnetic stimulation
SD
: standard deviation
SN: salience network
TANOVA: topographical analysis of variance
VSS: visual snow syndrome
woM: without migraine

## 1 Introduction

Visual snow syndrome (VSS) is a complex disorder affecting visual, salience, and attentional networks, with probably shared underlying neural mechanisms with migraine and tinnitus (Klein and Schankin 2021). VSS presents with various visual and nonvisual symptoms in individuals without other ophthalmologic or neurologic findings (Schankin et al. 2014). The primary symptom is the constant perception of flickering dots across the visual field. VSS is diagnosed (ICHD‐3, 2018) when patients report at least two additional symptoms such as palinopsia (persistent or recurring after images), photophobia (light aversion), nyctalopia (difficulty seeing in dim light), or enhanced entoptic phenomena like eye floaters. The diagnosis excludes cases caused by migraine aura or any other specific disorder. However, migraine headaches and tinnitus are common comorbidities (Schankin et al. 2014). VSS is estimated to affect about 2.2% of the population (Kondziella et al. 2020), diagnosis relies on patient reports, and treatment is lacking (Eren and Schankin 2020).

The syndrome involves multiple brain systems including the limbic system and parts of the parietal lobe responsible for visual, auditory, emotional, and cognitive processing (Schankin et al. 2020). The temporal dynamics within large scale networks typically changes within milliseconds. The dynamic is therefore best captured by methods with high temporal resolution like electroencephalography (EEG). Task‐based EEG studies are often used to investigate specific (ab)normal states of sensory processing but are not suitable to investigate and describe broader baseline changes (Li et al. 2022). In our study, microstate analysis of resting‐state EEG was used to compare the spatio‐temporal dynamics of large‐scale networks in VSS and a matched control group.

Microstates are short, distinct global states of brain processes that coalesce into conscious perception, which can be attributed to spontaneous mental activity (Khanna et al. 2015). Microstates have been considered the basic building blocks or “atoms of thought” (Lehmann et al. 1998). Each microstate is associated with a specific type of mental process, and the collective interaction of these microstates might contribute to the overall state of consciousness (Khanna et al. 2015).

Microstates remain quasi‐stable for about 60–120 ms before abruptly transitioning into another state and are represented in the EEG as global patterns of scalp topographies (Khanna et al. 2015; Lehmann et al. 1987). A variation in the spatial configuration suggests that they are generated by different neural sources, indicating changes in global network activity (Michel and Koenig 2018). Several studies demonstrated that only a few microstates, mainly the well‐replicated four so‐called canonical microstate Classes A–D, account for a significant amount (∼80%) of the variance in resting‐state EEG activity and correlate with fMRI resting‐state networks (Michel and Koenig 2018). Anomalies in the dynamics of these resting‐state EEG microstates are exhibited, for example, in psychiatric disorders such as schizophrenia, depression, and panic disorder (Khanna et al. 2015).

In respect of migraine without aura (Li et al. 2022; Zhou et al. 2023) and tinnitus (Cai et al. 2018; Cao et al. 2020) altered microstate architecture is known. Anomalies in microstate Class C correlated with subjective severity of tinnitus (Zhang et al. 2023) and migraine (Li et al. 2022). Considering VSS as a network disorder with probably shared underlying neural mechanisms with migraine and tinnitus, we assumed that and tested whether microstate Class C is altered in VSS too. Furthermore, we explored if the findings of structural, metabolic, and connection alterations in visual areas in VSS (Schankin et al. 2014; Puledda et al. 2021) are related to microstate Class B dynamics in VSS because of its link to visual processing (Tarailis et al. 2024). In short, we hypothesized that patients with VSS have altered parameters (duration, occurrence, coverage, and syntax) in microstate Classes B and C compared to controls.

## 2 Methods

The study design was preregistered on OSF.io (https://osf.io/xpqhf). Additionally, exploratory analyses were conducted and declared as such. The data used in this study were selected by trained individuals and provided by the University Hospital Inselspital of Bern (CH). The selected data has previously been analyzed to address potential changes in the alpha activity recorded over occipital sites due to VSS (Klein et al. 2024). In the present study, we use microstate analysis, which exploits the full spatio‐temporal structure of the data without assuming a specific location of a potential effect. The study was approved by the Bernese cantonal ethics committee (Protocol 2019‐02370) and complied with the Declaration of Helsinki.

### 2.1 Subject Recruitment

All participants (age ≥18 years) were examined in the outpatient clinic of the University Hospital of Bern (CH), and data was collected from the EEG database of patients from the Sleep‐Wake‐Epilepsy Center of the Neurology department. We included all available diagnosed VSS patients and matched control patients who underwent EEG as part of their routine clinical examination and gave written informed consent for further analysis of their data. Matching was based on age, sex, medication, migraine status, and other comorbidities (see Table 2). VSS was diagnosed by board‐certified neurologists who are experts in the field (A.K., C.J.S.). Patients with pathological EEGs (general or moderate or severe focal slowing, epileptiform discharges), specific psychiatric disorders (psychosis, schizophrenia, bipolar disorder, drug intoxication), major structural lesions, or neurological disorders were excluded. Patients with nonspecific white matter lesions, on the other hand, were included because this is commonly observed in individuals with migraine (Kruit et al. 2010). Patients with migraine were not in the ictal phase, although one control patient reported a mild headache. Two eligible patients were occasional cannabis users and were balanced between groups. One suitable patient had to be excluded because of lack of a general consent after preregistration, so we ended up including 21 participants per group rather than 22 as preregistered.

### 2.2 EEG Data Acquisition

EEG data were recorded with a NicoletOne system (Viasys Neurocare, Madison, WI, USA) in the EEG laboratory of the University Hospital of Bern with a sampling rate of 250 samples per second for 20 min. Twenty‐five Ag/AgCL bridge electrodes (Fz, Fp2/Fp1, F4/F3, F8/F7, F9/F10, C4/C3, Cz/Pz, T4/T3, T6/T5, T9/T10, P4/P3, P9/P10, and O2/O1), were used according to the standardized IFCN array (Seeck et al. 2017). Patients were asked to close their eyes, and it was regularly checked if they were awake. EEG segments with eyes closed were manually selected, and no subject was excluded due to artifacts.

### 2.3 EEG Preprocessing and Microstate Analysis

We performed all preprocessing in MATLAB (v.R2022b) using the EEGLAB toolbox (Delorme and Makeig 2004) (v.2022.1) and wrote scripts for each step to automate preprocessing. Eight artifact‐free epochs of 20 s from the resting‐state EEG data with eyes closed per person were selected (total = 159,998 s/subject). To compute microstates, we used the EEGLAB Microstate Analysis (v1.2). An overview on microstate analysis can be found in Michel and Koenig (2018), and details on our analysis are listed in Table 1. Briefly, preprocessed data is used to calculate the global field power (GFP) over time from the frequency range of interest (2–20 Hz). Then the data of all electrodes are compiled into global patterns of scalp potential topographies at each time point, with the highest signal‐to‐noise ratio at the peaks of the GFP, allowing specific maps to be generated. An algorithm then clusters the microstate maps to a specified number of microstate classes which must be sorted and backfitted to the EEG data (Khanna et al. 2015). In this study, we set four clusters and calculated their mean values for each group (grand means) and overall subjects (grand grand mean) and sorted the grand grand mean according to applicable norms. We backfitted the sorted grand grand mean to each group mean and these in turn to the corresponding individual microstates and then calculated the microstate parameters.

**TABLE 1:** Microstate analysis.

Steps		Implementation
(1)	Re‐referencing	Average
(2)	Filter	2–20Hz
(3)	Trimming recordings to equal length	129.996 s
(4)	Computing four individual microstate clusters	AAHC on all MS (infinitive) at GFP
(5)	Compute grand mean for four MS clusters	Mean microstates per group
(6)	Compute grand grand mean over clusters	Mean microstates over group means
(7)	Sorting grand grand mean	Implemented norms (NI2002)
(8)	Sorting and backfitting	Grand grand mean → grand mean → individual microstates
(9)	Quantify microstate parameters	On GFP peaks only, refusing truncated microstates

Abbreviations: AAHC, atomize and agglomerate hierarchical clustering; GFP, global field power (i.e., the strength of amplitude); MS, microstates.

### 2.4 Microstate Parameters

We computed six parameters to analyze the dynamics of microstates. The parameters were *duration*, that is, the average life span of a microstate during which it remains stable, in milliseconds. *Occurrence*, that is, the frequency of a microstate per second, *contribution*, that is, the amount of overall time spent in each microstate class, in percent. The *syntax* of microstates represents the transition probability from one microstate class to any other classes and can be interpreted as successive activation of different neural networks (Klein and Schankin 2021). We further compared the *global explained variance* of microstates between both groups, which is the total explained variance of a certain microstate and mean GFP, that is, the mean amplitude of a microstate in microvolt. The strength of GFP provides a reference free, global index of brain activity and is computed as the standard deviation across all electrodes (Koenig et al. 2011).

### 2.5 Statistical Analysis and Procedure

All data were analyzed using R studio (v1.2.5033, 2019). We conducted statistical comparisons with a *p* value of *p* < 0.05 and applied Bonferroni correction to account for multiple comparisons when needed. Values are expressed as mean (*M*) ± standard deviation (SD). We measured between‐group differences in microstate parameters (duration, occurrence, and coverage) with three separate mixed analyses of variance (ANOVAs), where the group was a between‐participants factor (VSS vs. control), and microstate class (A vs. B vs. C vs. D) was a within‐participants factor. Two‐tailed testing was used to assess differences between the groups. A priori assumptions of mixed ANOVAs, that is, sphericity, homoscedasticity, normality assumption, and outlier detection were tested for each parameter. In case of sphericity violation, a Greenhouse–Geisser correction was applied. For syntax analysis, the relative percentage of each possible transition from one microstate to another was calculated. We tested the significance of differences between groups using two‐sample Bonferroni‐corrected *t* tests for each of the 12 pairs of microstate transitions and post‐hoc test for significant effects. A topographical analysis of variance (TANOVA) was computed in RAGU via the toolbox MICROSTATELAB (v1.0) to compare microstate topographies between VSS group and controls. After normalizing the data, TANOVA averages the scalp potential topographies for each group and tests for significant differences between the topographic distribution of neural activity using randomization techniques (Koenig et al. 2011). No statistical power calculation was conducted before the study. However, VSS is a rare condition, and acquiring a large sample‐size is not feasible.

## 3 Results

### 3.1 Subjects and Microstates

**FIGURE 1:**
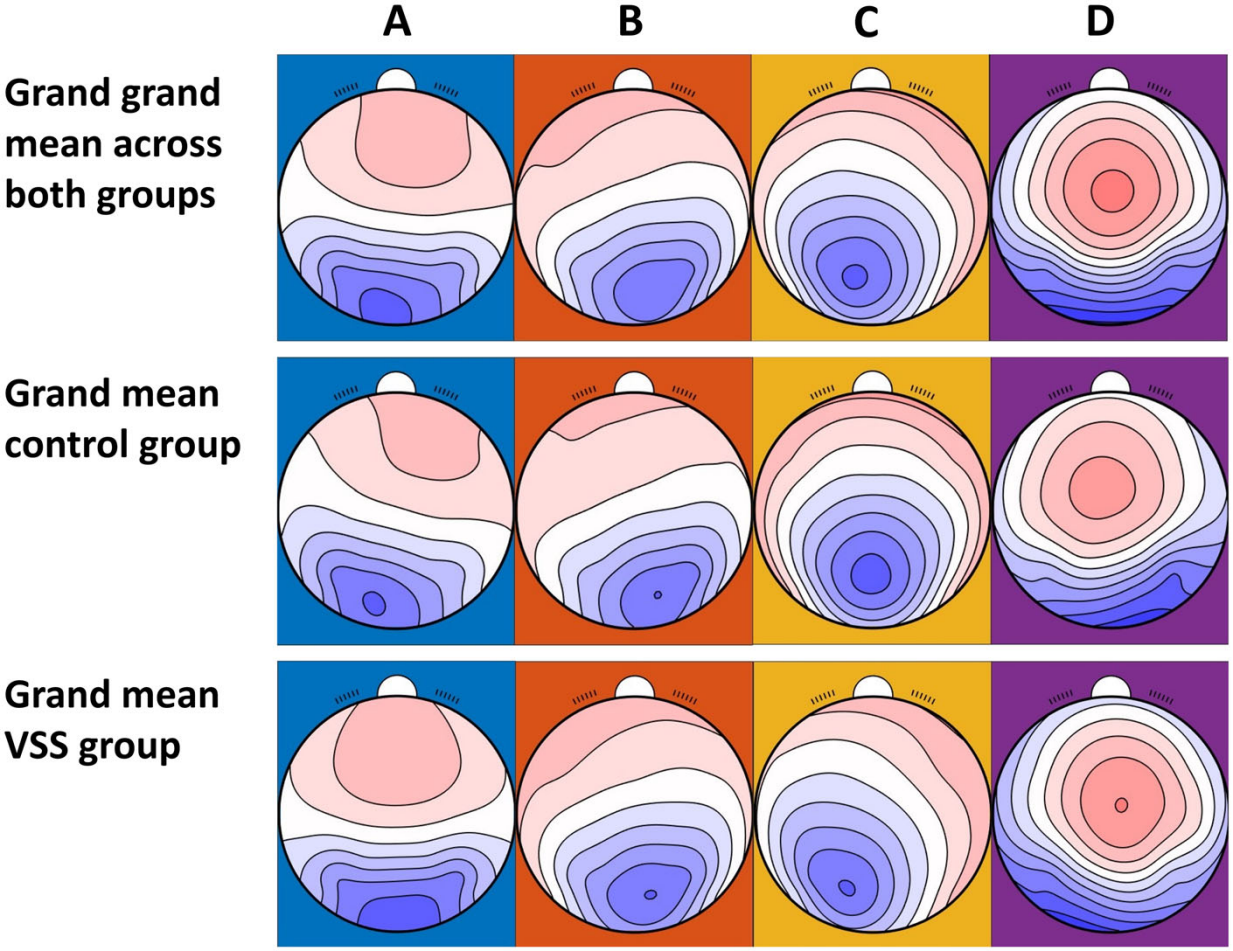
Mean microstates of both groups (*N* = 42). Microstate topographies of four microstate Clusters A–D. Top row: Grand grand mean across all subjects (*N* = 42), sorted by applicable norms; middle row: Grand mean across control group (*n* = 21), sorted by grand grand mean; bottom row: Grand mean across subjects with visual snow syndrome (*n* = 21), sorted by grand grand mean.

Twenty‐one patients with VSS (8 women; mean age 33 ± 9.56 years) were compared to 21 matched control subjects (8 women; mean age 33 ± 11.1 years). Distributions of the different demographics and symptoms are listed in Table 2. To calculate the parameter *duration*, we excluded one subject in the VSS group because outlier detection showed one extreme outlier (*M* + 3*SD; duration of microstate Class A = 136 ms) in this group. No other data was excluded or missing. The average microstate maps derived from both groups are shown in Figure 1. In line with previous literature (Michel and Koenig 2018), four different microstate topographies with their specific orientations were identified and labeled as A (right‐frontal left‐posterior), B (left‐frontal right‐posterior), C (anterior‐posterior), and D (fronto‐central extreme). The four computed microstates explained 79.1% (SD = 4.6) of the EEG data in controls and 77.5% (SD = 4.7) in VSS, and the variance did not differ between the two groups (*t*(165.92) = 1.96, *p* = 0.052, *d* = 0.61). Additionally, the TANOVA did not reveal differences between the two groups (*p* = 0.399) but between microstate classes and groups for Class A (*p* = 0.002), Class B (*p* = 0.014), Class C (*p* = 0.001), and Class D (*p* = 0.002).

**TABLE 2:** Demographic and clinical characteristics of subjects.

Characteristic	VSS (*n* = 21)	Controls (*n* = 21)
Male sex–no. (%)	13 (61.5)	13 (61.5)
Migraine–no. (%)	14 (66.6)	14 (66.6)
Age–mean ± SD (year)	33 ± 9.56	33 ± 11.1
Visual snow syndrome symptoms–no. (%)		
Visual snow–no. (%)	21 (100)	—
Photophobia–no. (%)	21 (100)	—
Enhanced entoptic phenomena–no. (%)	20 (95.2)	—
Palinopsia–no. (%)	14 (66.6)	—
Nyctalopia–no. (%)	13 (61.9)	—
Psychiatric comorbidities–no. (%)		
Depression/anxiety	8 (38.1)	5 (23.8)
ADHD	1 (4.7)	1 (4.7)
Functional neurological disorders	2 (9.5)	3 (14.3)
Eating disorder	1 (4.7)	0 (0)
Obsessive‐compulsive disorder	0 (0)	1 (4.7)
Previous drug abuse	0 (0)	1 (4.7)
Medication–no. (%)		
None	12 (57.1)	13 (61.9)
SSRI/SNRI/tricyclic antidepressant	4 (19)	5 (23.8)
Anticoagulation	0 (0)	2 (9.5)
Antihypertensive/antidiabetics	1 (4.7)	2 (9.5)
Antiseizure‐medication (Lamotrigine)	2 (9.5)	1 (4.7)
Calcium channel blocker (Cinnarizine)	0 (0)	1 (4.7)
Lisdexamfetamindimesilat	1 (4.7)	0 (0)
Benzodiazepines (intermittent)	1 (4.7)	0 (0)
Intermittent THC (intermittent)	1 (4.7)	1 (4.7)
Other comorbidities–no. (%)		
Chronic pain syndrome	1 (4.7)	0 (0)
Chronic leukemia	1 (4.7)	0 (0)
OSAS	0 (0)	1 (4.7)
Polycythemia vera	0 (0)	1 (4.7)

### 3.2 Microstate Parameters Between Groups (*N* = 42)

**FIGURE 2:**
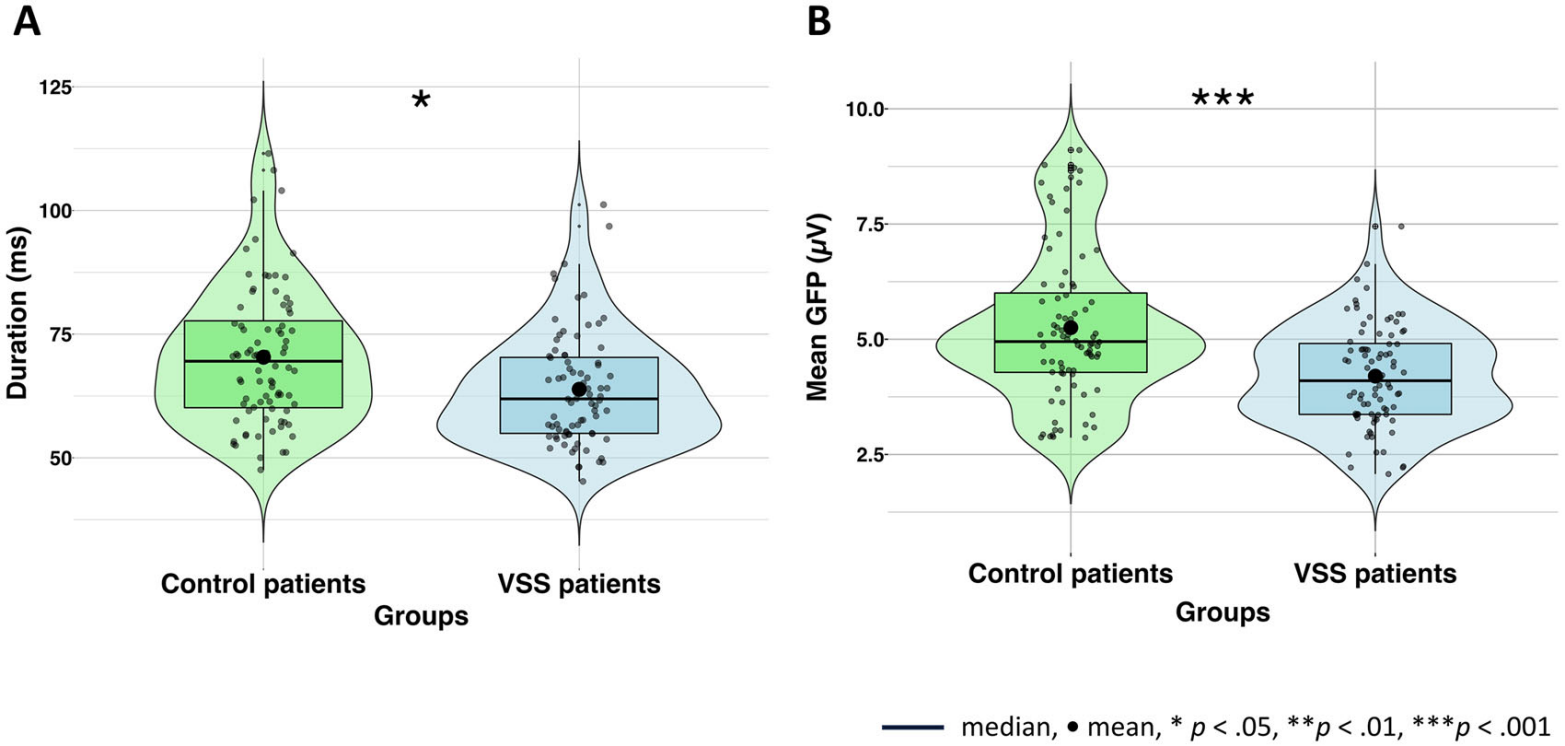
Findings of parameter *duration* (*N* = 41) and mean *global field power* (GFP) (*N* = 42). Significant main effects of group in life span and mean amplitude of microstates between visual snow syndrome patients and control patients. (A) A significant difference between both groups in overall lifespans of microstates in milliseconds. Parameter duration was analyzed with exclusion of one extreme outliner in patients with VSS (*n* = 20). (B) A significant difference between both groups in the overall amplitude of microstates.

A comparison of the different parameters of interest (POIs) between VSS and control group is listed in Table 3. Patients with VSS (*M* = 63.9, SD = 11.1) had overall shorter microstates (*t*(159.09) = −3.28, *p* = 0.001, *d* = −0.48) compared to controls (*M* = 70.3, SD = 13.6). A mixed ANOVA revealed main effects (see Figure 2a) of group (*F*(1, 39) = 5.22, *p* = 0.028, *η* ^2^ = 0.12) and microstate classes (*F*(3, 117) = 3.44, *p* = 0.0191, *η* ^2^ = 0.08), but no interaction between the two (*F*(3, 117) = 0.14, *p* = 0.933, *η* ^2^ = 0.00). The mean GFP differed between the two groups too (*t*(145.9) = −4.91, *p* < 0.001, *d* = −0.76), while patients with VSS had a lower mean amplitude (*M* = 4.199, SD = 1.1) compared to controls (*M* = 5.3, SD = 1.6) (a mixed ANOVA revealed main effects of group (*F*(1, 40) = 5.99, *p* = 0.019, *η* ^2^ = 0.13) (see Figure 2b), and microstate classes (*F*(3, 120) = 11.65, *p* < 0.001, *η* ^2^ = 0.23), but no interaction (*F*(3, 129) = 1.62, *p* = 0.188, *η* ^2^ = 0.04)). Bonferroni corrected pairwise *t* testing of microstate classes revealed no significant differences between the duration and amplitude for microstates. We did not find any group differences in parameters *occurrence* and *contribution* of the four microstates.

**TABLE 3:** Results of microstate parameters compared between patients with visual snow syndrome (VSS) and matched controls (*N* = 42, *n* = 21).

Parameter and microstate classes		VSS patients (*M* ± SD)	Control patients (*M* ± SD)
Duration (ms)			
MS A		70.07 ± 21.14	73.78 ± 15.65
MS B		65.38 ± 10.52	70.83 ± 13.07
MS C		62.30 ± 10.03	69.58 ± 9.55
MC D		59.40 ± 8.80	67.16 ± 16.13
Mean	*	64.29 ± 12.62	70.34 ± 13.60

Occurrence (/s)			
MS A		3.93 ± 0.66	3.60 ± 0.61
MS B		3.94 ± 0.80	3.41 ± 0.47
MS C		3.76 ± 0.91	3.72 ± 1.11
MC D		3.81 ± 0.84	3.49 ± 0.62
Mean		3.86 ± 0.80	3.56 ± 0.70

Contribution (%)			
MS A		28.11 ± 11.54	26.43 ± 6.64
MS B		26.03 ± 7.99	24.11 ± 5.29
MS C		23.38 ± 6.32	26.07 ± 9.19
MC D		22.48 ± 5.06	23.39 ± 6.51
Mean		25.00 ± 7.73	25.00 ± 6.91

Mean GFP (µV)			
MS A		4.37 ± 1.26	5.28 ± 1.71
MS B		4.29 ± 1.09	5.39 ± 1.76
MS C		4.07 ± 1.06	5.19 ± 1.47
MC D		4.05 ± 1.02	5.14 ± 1.67
Mean	***	4.20 ± 1.11	5.25 ± 1.65

*Note*: Significant main effects of groups in parameter *duration*, that is, the overall life span of microstates and *mean global field power*, that is, the mean amplitude of microstates.

Abbreviations: *M*, mean; SD, standard deviation.

^*^ *p* < 0.05, ^***^ *p* < 0.001.

**FIGURE 3:**
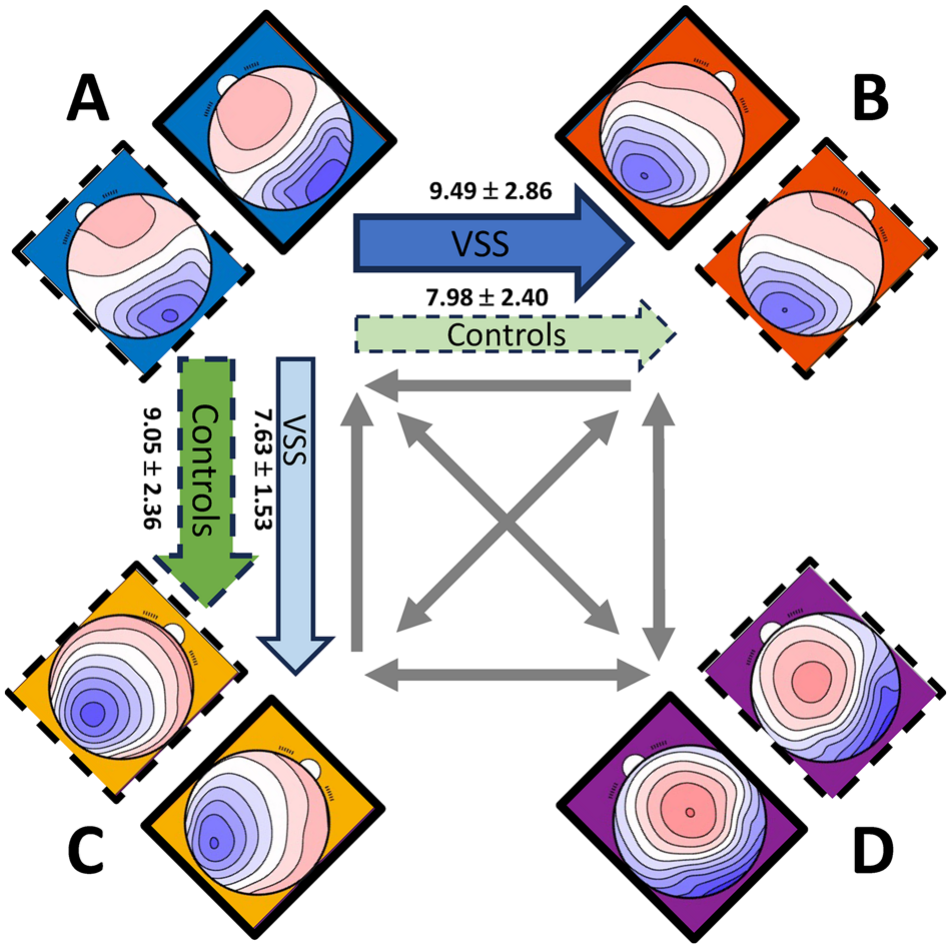
Observed relative transition percentages ± standard deviation from one microstate class to all other classes in both groups. Microstates A–D of VSS group have a solid frame, Microstates A–D of the control group are dashed framed. Colored arrows are significant, showing more frequent transitions for VSS (thick blue) from A to B and less transitions from A to C (thin blue) compared to controls (green dashed arrows). Gray arrows show no significant syntax differences between groups.

Figure 3 shows a schema of the observed transition probabilities from one microstate class to all other classes. Bonferroni corrected *t* tests between both groups revealed that patients with VSS had more transitions from microstate Class A to Class B (*t*(38.877) = 4.35, *p* = 0.001, *d* = 0.57) and fewer transitions from microstate Class A to Class C (*t*(32.074) = −3.67, *p* = 0.011, *d* = −0.71) compared to controls.

### 3.3 Explorative Analysis of Subgroups

**FIGURE 4:**
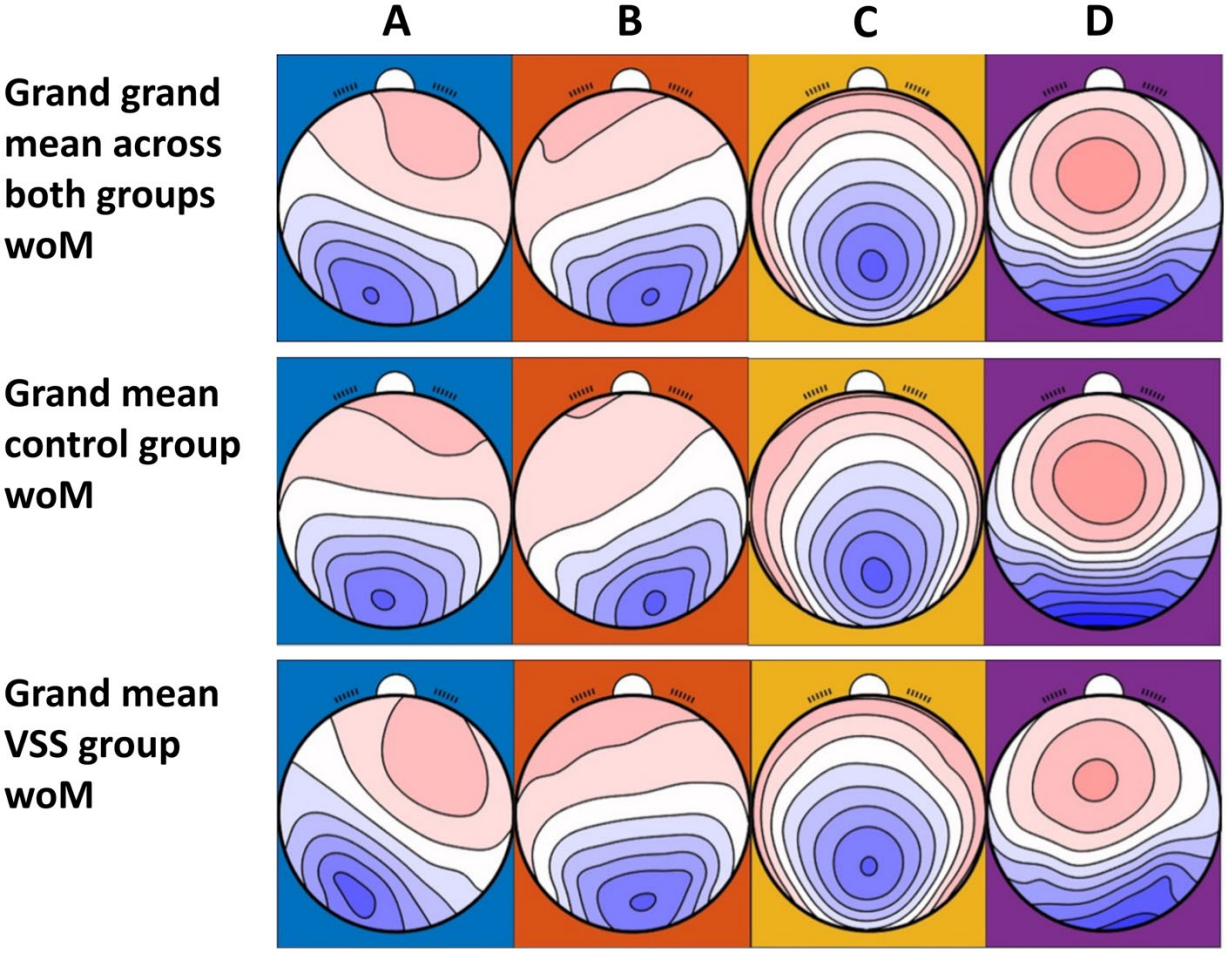
Mean microstates of both groups without migraine (woM) (*N* = 14). Microstate topographies of four microstate Clusters A–D for subgroup woM. Top row: Grand grand mean woM across all subjects woM (*N* = 14), sorted by implemented norms; middle row: Grand mean across control group woM (*n* = 7), sorted by grand grand mean woM; bottom row: Grand mean across subjects with visual snow syndrome woM (*n* = 7), sorted by grand grand mean woM.

We examined post‐hoc subgroup analyses with (*N* = 28, *n* = 14) and without migraine (woM) status (*N* = 14, *n* = 7) for exploratory insights. Contrary to woM, the subgroup with migraine showed no significant findings. Figure 4 shows the mean microstates of the subgroup woM, labeled according to previous work (Michel and Koenig 2018). The four computed microstates explained 80.34% (SD = 4.1) of the EEG data of control group woM and 78.38% (SD = 4.5) in patients with VSS woM, and both groups did not differ (*t*(53.42,) = 1.71, *p* = 0.093, *d* = 0.91).

### 3.4 Microstate Parameters in Subjects Without Migraine (woM, *N* = 14)

**FIGURE 5:**
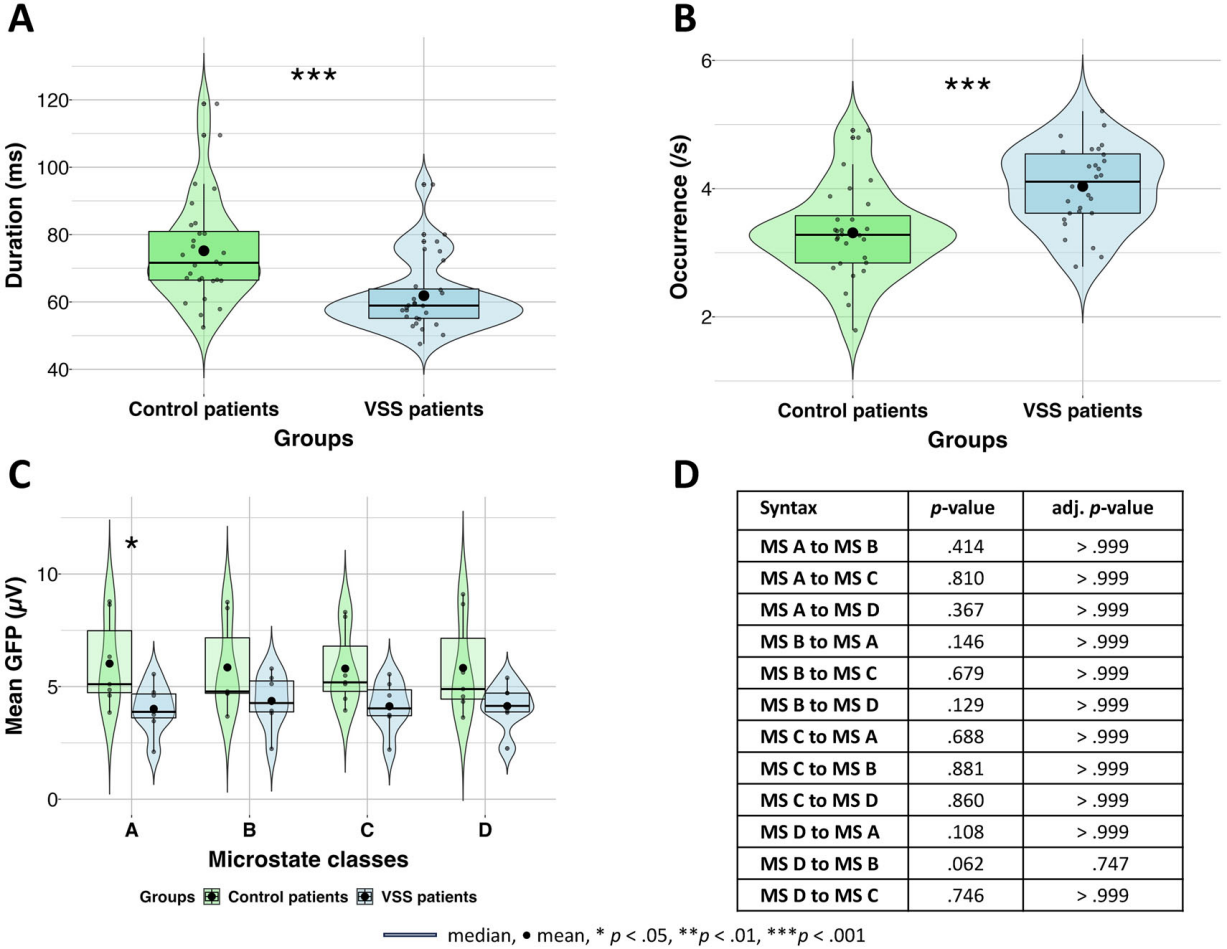
Findings in subgroup without migraine (woM) status (*N* = 14). (A) A significant difference between both groups in the overall duration of microstates, in milliseconds. (B) Significant difference between the two groups in the total frequency of microstates per second. (C) Mean global field power; significant difference between both groups in microstate Class A in its mean amplitude. (D) No significant results by comparing transition probabilities between both groups woM (*n* = 7).

Plots of significant POIs are shown in Figure 5a–c. Microstates were shorter (*t*(48.112) = 3.77, *p* < 0.001, *d* = 0.98) and occurred significantly more per second (*t*(53.14) = 4.01, *p* < 0.001, *d* = 1.07) in patients with VSS woM (duration in ms: *M* = 61.9, SD = 10.7; occurrence: *M* = 4.0, SD = 0.6) compared to controls woM (duration in ms: *M* = 75.2, SD = 15.4; occurrence: *M* = 3.3, SD = 0.7). Mixed ANOVAs revealed main effects of group in parameter *duration* (*F*(1, 12) = 8.02, *p* = 0.015, *η* ^2^ = 0.40) and *occurrence* (*F*(1, 12) = 8.43, *p* = 0.013, *η* ^2^ = 0.41). Parameter *mean GFP* revealed an interaction between groups and microstates classes (*F*(3, 36) = 3.25, *p* = 0.033, *η* ^2^ = 0.21). Microstate Class A had a significantly (*p* = 0.037) reduced mean amplitude in patients with VSS woM (*M* = 4.0, SD = 1.1) compared to controls woM (*M* = 6.0, SD = 2.0). Other ROIs did not reveal any significance.

## 4 Discussion

In this case–control study, we investigated the temporal dynamics of larg‐escale brain networks at rest by analyzing the spatial configuration of EEG microstates over time. Overall microstates were shorter in VSS patients regardless of migraine status compared to controls. We also found a lower mean amplitude in the VSS group and divergent transition probabilities for microstate Class A. The subgroup without migraine status showed a more frequent occurrence of microstates and a lower mean amplitude for microstate Class A in patients with VSS versus controls. However, we could not confirm our hypothesis of altered microstate parameters in microstate Classes B and C in patients with VSS compared to the control group. TANOVA showed that the four computed microstate Classes A–D differed significantly from each other, and thus altered networks are involved in the generation of microstates in VSS. These initial results suggest that neuronal markers based on microstate analysis represent a previously unused resource for understanding neurophysiological processes in VSS.

Given the diverse clinical presentation of VSS, it was crucial that the control group was consisted of individuals with matched conditions to eliminate potential confounding variables such as the impact of comorbidities (Al Zoubi et al. 2019; Damborská et al. 2019; Li et al. 2022; Zhou et al. 2023) or medications (Lei et al. 2022) on microstates. Our findings of spatial differences between microstates and groups indicate globally varying neuronal activity in VSS compared to control patients. The canonical microstate classes have specific topographies (Koenig et al. 2002) and are related to sensory processing (auditory and visual in Class A; visual in Class B), emotional and interoceptive (Class C), and attention‐related processing (Class D) (Tarailis et al. 2024). The altered topographies in VSS indicate that the sources generating the specific microstate classes might differ in the sense that less or additional neural populations are simultaneously active in resting‐state compared to controls. Moreover, the lower mean amplitude of microstates in VSS reflects fewer synchronized activities of assembled neural generators. These findings add evidence of temporal unorganized activity in large‐scale networks in VSS compared to controls. Besides changes in the topography and mean GFP, the duration of microstates is a crucial indicator of the stability of neural network activity. Our findings on general patterns of shortened microstates in VSS reflect unstable temporal dynamics in neuronal network activity. Taken together, these findings indicate that the stream of brain processes, that is, the collective interaction of microstates is altered, which may lead to the disturbing visual perceptions in VSS.

A disrupted global coordination of neural activity in VSS may also be manifested in an aberrant syntax. Microstate Class A showed anomalous transition probabilities to other classes in VSS and had a weaker mean amplitude in the subgroup without migraine. Microstate Class A was traditionally associated with auditory processing, but recent studies show a more heterogeneous picture and suggest its presence in visual network activity as well (Tarailis et al. 2024). Therefore, an altered microstate A syntax may reflect disruptions in sensory processing in VSS because of inaccurate sequential activated neural networks.

However, we could not show other specific alterations in microstates parameters between our groups, and a precise assignment of microstates to specific neuronal networks is not yet possible. Regarding common comorbidities of VSS, studies with migraine patients without aura demonstrated alerted parameters in resting‐state microstate Classes B, C, and D and aberrant transition probabilities between classes (Li et al. 2022; Zhou et al. 2023). In patients with tinnitus, aberrant dynamics of all resting‐state microstates were found, especially in Classes A and D (Cai et al. 2018; Cao et al. 2020). The fact that no specific microstate showed altered dynamics in VSS might reflect that higher‐order networks beyond the visual system are involved in the pathophysiology. This is also suggested by the clinical picture and the highly associated comorbidities (including migraine, tinnitus, affective symptoms, and chronic pain disorders) (Klein and Schankin 2021; Klein and Schankin 2021), making it difficult to identify consistent microstate patterns in VSS. Future studies with narrowly defined subgroups of VSS are needed to clarify this open question, especially regarding the variety of additional visual symptoms leading to diagnosis. Recent studies show a heterogeneous picture of large‐scale networks in generating specific microstates and suggest that certain microstates are associated with cognitive processes such as attention or sensory processing and integration (Tarailis et al. 2024). The specific assignment of microstates to cognitive functions is not consistent and requires further investigation.

The unstable manifestations of microstates are first temporal indicators of disrupted neural networks activity in VSS. This complements the results of fMRI studies showing altered connectivity within visual networks and with the default mode network (DMN), the dorsal attentional network (DAN), and salience network (SN) in patients with VSS (Puledda et al. 2021). The DAN and SN are both substantially involved in sensory processing, and imbalance leads to perceptual errors (Vossel et al. 2014; Menon and Uddin 2010). Thus, the aberrant global microstate manifestation may reflect deficits in information processing, sensory integration, and attention control, leading to the visual symptoms of VSS. Future studies on microstates in VSS using higher spatial resolution could shed light on how specific neuronal networks are temporally organized in VSS.

Our findings on microstates in VSS is important as they can be used to evaluate treatments. Modulation of the unstable large‐scale network activity, that is, aberrant syntax, short duration, and low mean GFP of microstates may be one way to alleviate the symptoms of VSS. For instance, initial studies on low‐frequency modulation have shown that the duration of the four canonical microstates can be extended by low‐frequency repetitive transcranial magnetic stimulation (rTMS) by increasing the stability of the EEG microstates (Qiu et al. 2020).

An important issue in the interpretation of EEG microstates is their potential role as biomarkers for neuropsychiatric disorders. Several studies have shown that patients with schizophrenia, depression, or other disorders may exhibit aberrant microstate patterns (Khanna et al. 2015). Studies on migraine without aura demonstrated an increased transition probability from A to B (Li et al. 2022) and a reduced one from A to C (Zhou et al. 2023). Our results show simultaneous occurrence of these patterns, which could not be shown in migraine alone. Therefore, findings of an aberrant syntax could serve as a potential biomarker for VSS, future studies are needed to replicate our findings. The challenge is to determine whether these deviations are a cause or a consequence of the underlying condition and how specific they are to VSS. Our findings of shorter lifespan and diminished mean amplitude of microstates strongly suggest unstable large‐scale network activity in VSS.

### 4.1 Limitations

There are limitations in this study. An exact matching on depression and anxiety was not possible, and no information of the tinnitus status in our cohort was available. Migraine phases prior and after the EEG recording are not documented. It is an open question how these comorbidities affected the results in VSS due to aberrant microstate patterns in these disorders (Cao et al. 2020; Al Zoubi et al. 2019; Murphy et al. 2020; Cao et al. 2016; Cai et al. 2018).

## 5 Conclusion

We used EEG microstate analysis to map neural dynamics of large‐scale network resting‐state activity in VSS. We demonstrated that the general manifestation of microstates and specific dynamics differed from those of matched control patients. Patients with VSS had a shorter duration and lower strength of microstates, indicating unstable, weaker, and varying neural network activity in this cohort. Microstate Class A showed abnormalities in transition behavior, suggesting an imbalance in sensory and interoceptive processing. This emphasizes that large‐scale network activity in VSS is imbalanced during resting‐state, complementing imaging studies in VSS and providing a potential treatment approach.

## Author Contributions

**Sarah A. Aeschlimann**: conceptualization, formal analysis, methodology, validation, visualization, writing–original draft. **Antonia Klein**: investigation, writing–review and editing, data curation, funding acquisition. **Frédéric Zubler**: writing–review and editing, resources. **Christoph J. Schankin**: funding acquisition, resources; writing–review and editing. **Matthias Ertl**: writing–review and editing, supervision, project administration, methodology, conceptualization.

## Conflicts of Interest

Christoph J. Schankin reports acting as a consultant, advisory boards, speaker, and travel support for/from Abbvie, Allergan, Almirall, Amgen, Eli Lilly, Grünenthal, Lundbeck, MindMed, Novartis, Pfizer, TEVA Pharmaceuticals, and part‐time employee at Zynnon. The other authors declare no conflicts of interest.

### Peer Review

The peer review history for this article is available at https://publons.com/publon/10.1002/brb3.70374.

## Data Availability

The data that support the findings of this study are available from the corresponding author upon reasonable request.
